# Intracytoplasmic Hemozoin (Malarial Pigment) in a Case of Severe, Aparasitemic *Plasmodium falciparum* Malaria

**DOI:** 10.4269/ajtmh.2011.10-0687

**Published:** 2011-04-05

**Authors:** Romualdo Grande, Carla Boschetti

**Affiliations:** Laboratorio Centrale Microbiologia, Fondazione IRCCS Cà Granda Ospedale, Maggiore Policlinico, Milano, Italy; U.O. Ematologia, Fondazione IRCCS Cà Granda Ospedale, Maggiore Policlinico, Milano, Italy

**Figure 1.**
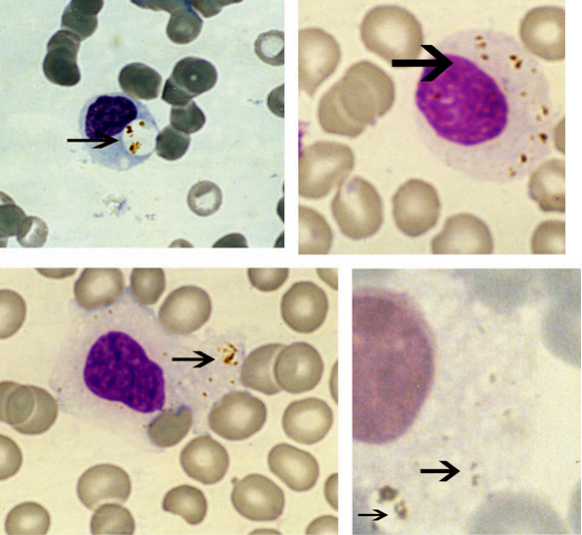
Intracytoplasmatic hemozoin granules (arrow) Giemsa thin films (100× magnification).

A 17-year-old girl from Togo with a β-thalassemia maior was seen at an emergency department with high fever, coma, and severe anemia (Hb < 3 g/dL). No malaria parasites were found on thin film and thick smears in the first few days of the infection. Despite the failure to observe *Plasmodium* intraerythrocytic forms in the bloodstream, many white blood cells showed brown, birefringent, intracytoplasmic inclusions (thick arrows) consistent with malarial pigment (hemozoin).The detection of intraleucocytic hemozoin with a negative malaria smear has to be considered as strongly suggestive of *Plasmodium falciparum* infection^1^ (Figure 1). Additional blood specimens were analyzed by a pan-human *Plasmodium* polymerase chain reaction and resulted positives. Further thin films and thick smears were prepared from fingerprick and venipuncture the next day, and they showed visible intraerythrocytic rings. The patient was treated with IV quinine and recovered completely.
